# Role of Diagnostic Hysteroscopy in Abnormal Uterine Bleeding, Its Histopathological Correlation, and Associated Metabolic and Endocrine Disorders: A Cross-Sectional Study

**DOI:** 10.7759/cureus.113814

**Published:** 2026-08-02

**Authors:** Nishat Fatima, Aparna Dwivedi, Manoj Kumar Prajapati, Prajakta G Chindhalore

**Affiliations:** 1 Obstetrics and Gynaecology, Government Medical College, Satna, IND; 2 Medicine, Government Medical College, Satna, IND; 3 Obstetrics and Gynaecology, N. K. P. Salve Institute of Medical Sciences and Research Centre and Lata Mangeshkar Hospital, Nagpur, IND

**Keywords:** abnormal uterine bleeding, diabetes mellitus, endometrial hyperplasia, endometrial polyp, histopathology, hypothyroidism, hysteroscopy

## Abstract

Introduction

Abnormal uterine bleeding (AUB) is an exceedingly prevalent complaint in gynecology and can be classified based on the underlying cause, such as a structural abnormality, an endocrine disorder, a metabolic disorder or abnormalities in the endometrium.

Objective

The objective of this study is to assess the hysteroscopic findings in women with AUB and assess the histopathology of these same women, while at the same time documenting the frequency of co-existing metabolic or endocrine disorders in the women with AUB.

Methods

This hospital-based cross-sectional study was carried out among 90 women with AUB attending a tertiary care center from April 2020 to April 2022. Consecutive eligible women underwent diagnostic hysteroscopy, followed by endometrial sampling and histopathological examination. Clinical data, metabolic and endocrine disorders, hysteroscopic findings, and histopathological diagnoses were obtained. Histopathological examination was the reference standard for hysteroscopy as a diagnostic modality. Sensitivity, specificity, positive predictive value (PPV), and negative predictive value (NPV) were calculated.

Results

Participants had a mean age of 35.8 ± 8.6 years, and the majority were females aged 25-35 years. The most frequent endocrine disorder was hypothyroidism, which affected 10 of the women (11.1%). Diabetes mellitus and hypertension each affected four women (4.4%). Hysteroscopy of the uterine cavity identified endometrial polyps in 24 women (26.7%) and endometrial hyperplasia (abnormal uterine lining) in 21 (23.3%), making them the two most prevalent abnormalities. Histopathology confirmed the presence of 24 endometrial polyps (26.7%) and endometrial hyperplasia (22.1%) in 20 women. Hysteroscopy had high diagnostic accuracy, with sensitivities of 91.7% and 88.0%, and specificities of 97.0% and 91.8% for endometrial polyps and endometrial hyperplasia, respectively. There were no complications due to the procedure.

Conclusions

The use of hysteroscopy to evaluate patients with AUB provided a well-defined correlation between histopathological and hysteroscopic findings. Hysteroscopy was able to accurately identify the most commonly diagnosed problems associated with AUB, such as endometrial polyps and endometrial hyperplasia. It was also able to identify the most frequently diagnosed hormonal disorder: hypothyroidism.

## Introduction

Abnormal uterine bleeding (AUB) describes menstrual bleeding that is excessive or inadequate in volume, irregular in its duration, and does not follow a prescribed cycle and has a profound impact on the patient's quality of life [1]. AUB is a common reason for patients to present for evaluation by their gynecologist or other physician and requires extensive workup and testing to identify the underlying diagnoses and ultimately determine the appropriate treatment. The causes of AUB are multifactorial and include abnormalities of the uterus, such as fibroids, dysregulation of ovulation, endometrial conditions, systemic diseases, or are secondary to medications prescribed for a non-gynecologic condition. The International Federation of Gynecology and Obstetrics (FIGO) developed the Polyp, Adenomyosis, Leiomyoma, Malignancy and Hyperplasia-Coagulopathy, Ovulatory Dysfunction, Endometrial, Iatrogenic, and Not Yet Classified (PALM-COEIN) classification as a standardized system for evaluating AUB [2]. Disorders of the endocrine system can cause menstrual disorders and abnormalities as well as pathologies of the endometrium. Thyroid hormones impact the regulation of the hypothalamic-pituitary-ovarian axis, in which there is a disruption in ovulation, irregular menses, or AUB as a result of hypothyroidism [3]. Hypothyroidism has been associated with an increased risk for menstrual abnormalities and has been proposed as a contributing factor for abnormal endometrial proliferation by hormonal dysregulation [3]. Similarly, metabolic diseases (e.g., diabetes and hypertension) have been associated with increased risk of endometrial hyperplasia and carcinoma of the endometrium, due to insulin resistance, chronic inflammation, and alterations in estrogen metabolism [4]. An endometrial assessment is vital for evaluating women with AUB. Endometrial sampling is an important tool for determining endometrial histopathology [5]. Guidelines for clinical evaluation of AUB have been developed recently and emphasize a thorough evaluation of women with AUB to help appropriately diagnose and manage the underlying pathology [6]. Blind sampling techniques may not detect focal or irregular intrauterine pathology, leading to an insufficient diagnosis of endometrial pathology. Hysteroscopy is a surgical technique that allows for direct visualization of the endometrium, including any abnormalities in and around the uterus, and can be combined with biopsy of suspected abnormalities in order to have a thorough diagnosis. Hysteroscopy has been shown to have improved effectiveness in diagnosing endometrial polyps, hyperplasia, submucosal leiomyomas, and other abnormalities inside the uterus compared to traditional diagnostic techniques [7]. Correlating hysteroscopic findings with histopathological evaluation provides further evidence of an abnormality and enhances the clinician's ability to create an appropriate management plan for a woman with AUB.

The purpose of this research study is to evaluate the role of diagnostic hysteroscopy in detecting focal intracavitary lesions, particularly endometrial polyps, among women with AUB and to correlate hysteroscopic observations with histopathological findings.

## Materials and methods

A hospital-based cross-sectional study was carried out in the Department of Obstetrics and Gynaecology, Mahatma Gandhi Memorial (MGM) Medical College and Associated Hospitals in Indore, Madhya Pradesh, India, from April 2020 to April 2022 with Institutional Ethics Committee approval under EC/MGM/March-19/03. Prior to enrollment, all participants were provided with written informed consent, and the confidentiality of the participants' information was maintained at all times throughout the study. Consecutive eligible patients who met the selection criteria and were willing to participate were enrolled in the study. A total of 90 women were included and underwent diagnostic hysteroscopy followed by endometrial sampling for histopathological examination. Women who were married, were above 25 years old, and were willing to provide informed consent, and who had an abnormal pattern of uterine bleeding were included in the study. Women with cervical cancer, vulvar cancer, vaginal cancer, acute pelvic inflammatory disease, women currently taking hormonal therapy, women experiencing acute profuse uterine bleeding needing emergency intervention, women with recent uterine perforation, and women who do not want to participate were excluded from the study.

Data sources and variables

Data were derived from clinical examination, medical history, patient history, interviews with participants, hysteroscopic examination, and histopathologic examination. Demographic variables included age, parity, symptom duration, menstrual history, body mass index (BMI), and other clinical information relevant to their care or treatment plan. Evidence of doctor-diagnosed or undiagnosed metabolic or endocrine disorders, such as hypothyroidism, diabetes mellitus, and hypertension, was documented through patient history, memos from physicians who treated or evaluated this patient, and ongoing treatment information regarding these conditions. Hysteroscopic examination data included the following: proliferative endometrium; secretory endometrium; endometrial hyperplasia; endometrial polyp; submucous myoma; atrophic endometrium; retained intrauterine device (IUD); and many other types of intrauterine lesions that could be discovered during this examination. Histopathological data included as follows: proliferative endometrium; secretory endometrium; endometrial hyperplasia; endometrial polyp; endometritis; atrophic endometrium; submucous myoma; and irregular ripening, among others. The standard for diagnostic performance of hysteroscopy was histopathological examination.

Statistical analysis

The data were entered into Microsoft Excel (Microsoft® Corp., Redmond, WA) and analyzed using Statistical Package for the Social Sciences (SPSS) version 25.0 (IBM Corp., Armonk, NY). Continuous variables were expressed as a mean with a standard deviation, while categorical variables were presented as frequency or percentage tables, to determine the diagnostic performance of hysteroscopy (sensitivity, specificity, positive predictive value (PPV), negative predictive value (NPV) against a reference standard of histopathological examination) and by using the descriptive statistics method of analysis to assess the demographic characteristics, metabolic and endocrine disorders, hysteroscopic findings and histopathological diagnoses.

## Results

A total of 90 women presenting with AUB were included in the study. The mean age of participants was 35.8 ± 8.6 years. Most women belonged to the reproductive age group and were multiparous.

Table 1 summarizes the demographic and clinical characteristics of the study population. Women aged 25-35 years constituted 52 participants (57.8%). Most participants were multiparous, accounting for 68 women (75.5%), while 81 women (90.0%) had a normal BMI.

**Table 1:** Demographic and clinical characteristics of study participants (n = 90) BMI: body mass index

Variable	Frequency (%)/mean ± SD
Mean age, years	35.8 ± 8.6
Age 25-30 years	26 (28.9%)
Age 30-35 years	26 (28.9%)
Age 35-40 years	12 (13.3%)
Age 40-45 years	11 (12.2%)
Age ≥45 years	15 (16.7%)
Duration <6 months	41 (45.5%)
Duration 6-12 months	25 (27.8%)
Duration >1 year	24 (26.7%)
Multiparous	68 (75.5%)
Nulliparous	22 (24.5%)
Body mass index 18-25 kg/m²	81 (90.0%)
Body mass index >25 kg/m²	9 (10.0%)

The distribution of the various metabolic and endocrine disorders among participants in the study is shown in Table 2. Ten (11.1%) females experienced hypothyroidism, which was the most frequently occurring associated illness, while four (4.4%) had diabetes mellitus and four (4.4%) had hypertension. The number of females without documented metabolic or endocrine disorders was 72 (80.0%).

**Table 2:** Associated metabolic and endocrine disorders (n = 90)

Disorder	Frequency (%)
Hypothyroidism	10 (11.1)
Diabetes mellitus	4 (4.4)
Hypertension	4 (4.4)
None	72 (80.0)

Table 3 outlines the evaluated hysteroscopic findings. Of note are endometrial polyps (24 patients, 26.7%), followed by proliferative endometrium (22 patients, 24.4%) and endometrial hyperplasia (21 patients, 23.3%). Other types of hysteroscopic findings included submucosal myoma, atrophic endometrium, absence of IUD, and a "Swiss cheese" endometrium.

**Table 3:** Hysteroscopic findings in women presenting with abnormal uterine bleeding (n = 90) IUD: intrauterine device

Finding	Frequency (%)
Proliferative endometrium	22 (24.4)
Secretory endometrium	12 (13.3)
Endometrial hyperplasia	21 (23.3)
Endometrial polyp	24 (26.7)
Submucous myoma	7 (7.8)
Atrophic endometrium	8 (8.8)
Missing IUD	6 (6.6)
Swiss cheese appearance	2 (2.2)

The histopathological results are shown in Table 4. Among the 90 women studied, 24 (26.7%) had endometrial polyps, and 20 (22.1%) had endometrial hyperplasia; 16 (17.8%) had a proliferative type endometrium, and 12 (13.3%) had a secretory type endometrium. In summary, the majority of histopathological results for the women in this study were benign endometrial lesions.

**Table 4:** Histopathological findings following endometrial sampling (n = 90)

Histopathological diagnosis	Frequency n (%)
Proliferative endometrium	16 (17.8%)
Secretory endometrium	12 (13.3%)
Endometrial hyperplasia, total	20 (22.1%)
Simple hyperplasia	12 (13.3%)
Cystic glandular hyperplasia	3 (3.3%)
Complex hyperplasia	5 (5.5%)
Endometrial atrophy	8 (8.8%)
Submucous myoma	6 (6.6%)
Endometritis	2 (2.2%)
Irregular ripening	3 (3.3%)
Endometrial polyp	24 (26.7%)

The diagnostic capabilities of hysteroscopy, as measured against histopathological reports, are illustrated in Table 5. The hysteroscopy was highly accurate in determining whether a specimen had secretory or atrophic endometrium, with both its sensitivity and specificity equal to 100%. In addition, there were also high-hysteroscopy diagnostic parameters for identifying endometrial polyps (sensitivity of 91.7% and specificity of 97.0%), and endometrial hyperplasia (sensitivity of 88.0% and specificity of 91.8%). There were no complications related to hysteroscopy throughout this research study.

**Table 5:** Diagnostic accuracy of hysteroscopy compared with histopathological examination NPV: negative predictive value; PPV: positive predictive value

Lesion	Sensitivity (%)	Specificity (%)	PPV (%)	NPV (%)
Proliferative endometrium	62.5	100	100	92.5
Secretory endometrium	100	100	100	100
Endometrial polyp	91.7	97.0	91.6	96.9
Atrophic endometrium	100	100	100	100
Submucous myoma	100	97.6	71.4	100
Endometrial hyperplasia	88.0	91.8	71.4	97.1

## Discussion

This research examined hysteroscopic findings and their histological correlations among women presenting with AUB. In addition, the study examined metabolic and endocrine disorders that may be associated with persistent AUB. The findings of this study show that most of the women studied are of reproductive age. Also, hypothyroidism was the most common endocrine disorder, and hysteroscopic results were a very accurate method for assessing endometrial pathology as compared with histological exams. The majority of participants in this study were in the 25- to 35-year-old age bracket, consistent with previous studies showing that this age group is most likely to have AUB due to hormone fluctuations, ovulatory dysfunction, and an abnormal endometrium [8]. Hospital-based studies examining women with AUB report similar age distributions [9].

Hypothyroidism was the most common endocrine disorder discovered (10 women, 11.1%). More specifically, thyroid hormones play a key role in maintaining normal reproductive function. If thyroid hormone levels are abnormal, menstrual symptoms may result from disturbances in the hypothalamic-pituitary-ovarian axis [3]. Importantly, previous studies have shown that women who have thyroid issues also tend to have higher rates of menstrual disruption and AUB [10]. The most common hysteroscopic abnormality found during this study was endometrial polyps. The second was endometrial hyperplasia. These data are consistent with other researchers [11]. Similarly, recent work by Guin et al. supports the need for both direct inspection and targeted biopsies in women suspected of having AUB [12].

Histopathological data supported the presence of a significant number of benign lesions in the endometrium. Endometrial polyps and hyperplasia were the most frequently found benign lesions. Importantly, these lesions can become malignant or pre-malignant if allowed to persist [13]. The congruence between hysteroscopic and histopathological findings confirms the effectiveness of hysteroscopy in evaluating the endometrium [14]. The clinical utility of hysteroscopy was confirmed in this study, as it was highly accurate for diagnosing endometrial polyps, endometrial hyperplasia, an atrophic endometrium, and submucous fibroids. Again, histopathological findings confirmed the reliability of hysteroscopy to diagnose endometrial disease. There are reports of contemporaneous systematic reviews and studies that have corroborated the diagnostic accuracy of hysteroscopy for women with AUB [15,16]. The lack of complications during hysteroscopy in this study supports the general safety and effectiveness of this diagnostic method when performed by qualified practitioners.

Strengths and limitations

There are many strengths associated with this research, including the evaluation of hysteroscopic measures relative to their histopathological counterparts and the inclusion of data on other medical conditions related to hormonal and metabolic regulation among women with AUB. However, because this was a single-center study with a limited patient population, the generalizability of the findings may be limited. Moreover, due to the limited number of participants with other comorbidities (e.g., diabetes and hypertension), a complete analysis of subgroup comparisons was not possible.

## Conclusions

Patients who present with AUB are frequently found to have underlying endocrine or metabolic conditions that may contribute to this symptom. The most prevalent endocrine condition identified in the current study is thyroid dysfunction or hypothyroidism. Hysteroscopy has been reported to have a high diagnostic accuracy for endometrial pathology and strong histopathological concordance in detecting endometrial polyps and hyperplasia. Hysteroscopic evaluation is considered an effective and safe method for assessing AUB. Patients may benefit from integration of metabolic and endocrine evaluations with hysteroscopic evaluation to facilitate a complete assessment of patients and a more individualized approach to the management of these patients.
